# Atypical Ductal Hyperplasia at the Margin of Lumpectomy Performed for Early Stage Breast Cancer: Is there Enough Evidence to Formulate Guidelines?

**DOI:** 10.1155/2012/297832

**Published:** 2012-12-04

**Authors:** Jennifer L. Baker, Farnaz Hasteh, Sarah L. Blair

**Affiliations:** ^1^Division of General Surgery, Department of Surgery, UCSD Medical Center, University of California, San Diego, CA 92103, USA; ^2^Division of Surgical Pathology and Cytopathology, Department of Pathology, UCSD Medical Center, University of California, San Diego, CA 92093, USA; ^3^Division of Surgical Oncology and Breast Surgery, Department of Surgery, UCSD Moores Cancer Center, University of California, San Diego, CA 92093, USA

## Abstract

*Background*. Negative margins are associated with a reduced risk of ipsilateral breast tumor recurrence (IBTR) in women with early stage breast cancer treated with breast conserving surgery (BCS). Not infrequently, atypical ductal hyperplasia (ADH) is reported as involving the margin of a BCS specimen, and there is no consensus among surgeons or pathologists on how to approach this diagnosis resulting in varied reexcision practices among breast surgeons. The purpose of this paper is to establish a reasonable approach to guide the treatment of ADH involving the margin after BCS for early stage breast cancer. *Methods*. the published literature was reviewed using the PubMed site from the US National Library of Medicine. *Conclusions*. ADH at the margin of a BCS specimen performed for early stage breast cancer is a controversial pathological diagnosis subject to large interobserver variability. There is not enough data evaluating this diagnosis to change current practice patterns; however, it is reasonable to consider reexcision for ADH involving a surgical margin, especially if it coexists with low grade DCIS. Further studies with longer followup and closer attention to ADH at the margin are needed to formulate treatment guidelines.

## 1. Introduction

Breast conserving surgery (BCS) is a widely accepted treatment option for early-stage breast cancer based on several prospective, randomized trials that demonstrate equivalent survival after BCS compared to that after mastectomy [1–7]. The recent 20-year followup of the NSABP B-06 trial recognizes an increased risk of ipsilateral breast tumor recurrence (IBTR) after BCS; however, this risk is decreased with the addition of whole breast irradiation and obtaining negative margins [1]. While the validation of BCS requires that a negative margin be obtained, there is still no consensus definition of what constitutes an adequate negative margin width resulting in marked variation in BCS reexcision practices among surgeons [8–10]. While all agree it is not appropriate to have tumor cells involving the inked margin, there is no compelling or consistent evidence to indicate how widely free a margin should be [11–15]. Accordingly, margin width alone may not be sufficient to prove adequacy of excision and qualitative and quantitative pathologic characteristics of the cells within the margins may be important to consider [16]. One of those factors is proliferative lesions and in particular atypical ductal hyperplasia (ADH) at the margin of BCS specimens [17]. Interestingly, despite the high volume of studies investigating the question of adequate margin width, there is a paucity of studies that address the pathological characteristics of the cells within the margin. Not surprisingly, in the setting of no evidence-based guidelines, neither pathologists nor surgeons know what to do with the diagnosis of ADH involving a margin of a BCS specimen performed for early-stage breast cancer [18–20]. The purpose of this paper is to address the significance of a diagnosis of ADH at the margin and evaluate if there is evidence to guide the surgical decision for reexcision.

## 2. Methods

We searched the PubMed database for studies evaluating atypical ductal hyperplasia published in English with no date range qualification. We searched Medical Subject Headings (MESH), titles, and abstracts for the terms atypical ductal hyperplasia (ADH), breast conserving therapy (BCS), ADH at margin, proliferative lesion of the breast, ipsilateral breast tumor recurrence (IBTR). All major studies evaluating margin status and IBTR after BCS were evaluated for purposeful attention to ADH at the margin. 

## 3. Results

### 3.1. Atypical Ductal Hyperplasia: A Histopathologic Prospective

Atypical ductal hyperplasia (ADH) is an atypical proliferative lesion that falls in between the continuum from normal hyperplasia to low grade ductal carcinoma in situ (LG-DCIS). There is currently no general agreement on quantitative versus morphologic criteria to separate ADH from DCIS. However, some define ADH as the cells with morphologic characteristics of LG-DCIS (i.e., a cribiform or micropapillary proliferation of uniform cells with low-grade nuclei) with partial involvement of the terminal duct lobular unit (TDLU) or involvement in less than 2 separate duct spaces or less than 2 mm in aggregate diameter [17, 24–26]. This is a purely quantitative and not a biologic distinction [27, 28], and as such it can be a subjective diagnosis in practice. A recent review acknowledged differentiation between ADH and LG DCIS is one of the most challenging areas in diagnostic pathology [28]. Consequently, the diagnosis has proven vulnerable to a large interobserver variability even between highly trained breast pathologists in optimal conditions as demonstrated in multiple studies [18, 21–23].  Table 1 demonstrates the differing rates of agreement between pathologists observed in 3 well-known studies that investigated concordance rates among pathologists interpreting proliferative lesions. Rosai [21] and Jain et al. [23] found complete agreement amongst pathologists deciphering ADH from DCIS only 0% and 32% of the time, respectively. Schnitt et al. [22] found slightly improved agreement after diagnostic criteria for proliferative lesions was provided to the pathologists prior to slide interpretation, but still only found all pathologists agreed <60% of the time when diagnosing a lesion as DCIS versus ADH. A recent study by Ghofrani et al. [18] is consistent with this phenomenon. They sent a single diagram depicting a partially involved duct adjacent to unequivocal DCIS to 230 pathologists known for their expertise in breast pathology and asked them to interpret the diagrammatic representation. When looking at the exact same lesion, 56.5% of the pathologists considered it ADH and 43.5% interpreted it as DCIS.

### 3.2. Significance of ADH

It is well established in the literature and in practice that the diagnosis of ADH on core needle biopsy (CNB) necessitates a subsequent excisional biopsy [19]. This recommendation is based on the difficult pathologic distinction between ADH and LG DCIS especially in settings of small tissue samples, and also the fact that ADH on CNB is associated with a high degree of upstaging to in situ and invasive cancer on subsequent excisions at published rates varying between 24 and 45% [29]. Historically, ADH was considered only a risk factor of subsequent cancer conferring a 4-5x increased risk of invasive carcinoma in either breast [30]; however, recent studies have challenged this and provided some genetic and molecular evidence that ADH is a precursor to a low grade cancer [27, 31]. When ADH is diagnosed in the setting of a known DCIS or invasive, some pathologists report it as a distinct lesion from ADH diagnosed independent of a neoplastic lesion. In a study by Lennington et al. [32] that investigated patterns of DCIS, the authors found that ADH associated with DCIS located at the periphery of the lesion; thus, indicating that when ADH is at the margin of a BCS specimen, it likely represents the most peripheral extent of the neoplastic lesion. Goldstein agrees with this concept when he describes a foci of ADH identified near the margin of an excision specimen for DCIS or invasive carcinoma represents partial involvement of lobules by intraluminal neoplastic cells and is the farthest tentacular extension of low-grade intraductal carcinoma [17]. 

### 3.3. ADH at the Margin of a BCS Specimen for Early Stage Breast Cancer: Current Trends among Surgeons and Pathologists

Nizre et al. [19] conducted an important survey capturing the current management of breast borderline lesions. The survey was sent to members of the American Society of Breast Surgeons (ASBS).Responses from 477 surgeons were received and analyzed. Importantly, 337 of the respondents dedicated more than 50% of their practice to breast surgery and 50% were from academic, cancer center, or dedicated breast centers. When asked about how to manage a diagnosis of ADH within 1 mm of a BCS specimen, 61% favored no further surgery while 30% recommended selective reexcision. Interestingly, the amount of training affected response tendencies towards no further surgery. For example, among surgeons practicing at a cancer center, 80% would recommend no further surgery, 20% would recommend selective reexcision, and 0% recommended routine reexcision when ADH involved the margin. This is compared to private practice where 54% would recommend no further surgery, 40% would selectively reexcise, and 5% would routinely reexcise. This difference between groups was found to be statistically significant and the same significant trend favoring not to reexcise was seen amongst surgeons participating in weekly tumor boards and those trained in surgical oncology. Another questionnaire study involving 200 breast surgeons in the United Kingdom showed less variation in surgeon practices with 91% of respondents favoring no further surgery if there was ADH at the margin of excision, but both invasive and in situ disease were 10 mm clear of the margin [20]. 

There is a large practice variation amongst American pathologists as well as demonstrated in the study conducted by Ghofrani et al. [18], which was described earlier in our paper. In addition to classifying the proliferative lesion adjacent to DCIS as ADH or DCIS, the pathologists were also asked what to do with the lesion if it involved the margin of a BCS specimen. Regardless of whether the responders diagnosed the lesion as ADH or DCIS, the final impact on management was that 50.4% would recommend to reexcise based on the lesion being present at the margin while 47% would not reexcise. Of those that considered the lesion ADH, 37.7% recommended reexcision while only 28% of those who considered the lesion DCIS recommended to reexcise.

### 3.4. Studies Evaluating ADH at the Margin of BCS Specimens

There is a paucity of studies evaluating ADH at BCS margins. Only two studies were found in the literature that directly addressed the issue and they came to disagreeing conclusions. One study reported compelling evidence for reexcision if ADH involved the margin. It was a retrospective review at a single institution (Mt Sinai Medical Center, New York) that spanned 6 years (2000–2006) and sought to determine the rate of residual disease when reexcision was performed for ADH involving the margin of lumpectomies performed for ADH or early stage breast cancer [36]. They identified 44 lumpectomy specimens performed for ADH or early stage breast cancer where ADH involved the margin (at or within 1 mm). 27 of the 44 cases underwent reexcision and of the 27 that underwent reexcision and 26% had either DCIS or invasive disease. They included the diagnosis of the original lumpectomy to evaluate the rates of residual disease based on lumpectomy for ADH versus DCIS versus invasive disease. There were 7 lumpectomies for DCIS reexcised for ADH involving the margin and of those, 2 reexcision specimens had residual ADH (28.6%) and 4 had residual DCIS (57%). There were 2 lumpectomies for invasive disease reexcised for ADH involving the margin and both specimens had residual ADH, but no DCIS or invasive disease identified on reexcision specimens. Despite the small study sample, they concluded that ADH at the margin of a lumpectomy specimen is associated with a high rate of residual ADH or cancer, and they recommend reexcision in all patients with ADH involving the margin. 

A different study by Goldstein et al. [33] investigated whether ADH involving the margin of a BCS specimen predicted IBTR. He retrospectively reviewed the slides of 94 patients treated with local excision followed by radiation with particular attention to the presence of ADH at the margins on the slides of final excision specimen. At a median followup of 78 months, true recurrence (defined as recurring in the same area as the original lumpectomy) developed in 6 patients. DCIS and ADH within .2 cm of the margin was associated with recurrence, but there was no association with recurrence when ADH appeared alone. Importantly, all 6 true recurrences occurred in cases where either ADH alone, DCIS + ADH, or DCIS + cancerization of the lobules (COL) was involving the margins of the initial specimen. One limitation in this study is the short followup time which could be too short to detect recurrences in the specimens with only ADH at the margin as the interval to development of breast cancer in an ADH lesion is 8.2 years [31].

Greene et al. [35] performed an important study evaluating ADH at the margin of lumpectomies performed solely for ADH (none of lumpectomies evaluated were positive for malignancy). They identified 87 lumpectomies with margins positive for ADH. Of those with positive margins, none went on to develop a malignancy. A significant limitation of this study is a short followup of 26 months, and none of the lumpectomies with an initial diagnosis of malignancy were included in the study. 

Some clinicians have used a study performed by Fowble et al. [34] to support no reexcision for ADH at the margin [20]. The Fowble study evaluated the influence of proliferative lesions in the background benign breast tissue of BCS specimens performed for stage I and II breast cancer. In their study, they retrospectively reviewed the pathology slides of 460 BCS specimens and found that 99 out of 460 specimens contained background atypical ductal hyperplasia. The authors did not demonstrate an increased risk of IBTR with median followup 5.6 years in the specimens with ADH compared to those specimens without ADH. Unfortunately, this study did not specify where the ADH was located in the specimen and did not identify which specimens had ADH involving the margin. Rather, they just confirmed the presence of ADH anywhere in the specimen, and thus, it is not possible to make assumptions about recurrence rates in those specimens where the ADH did involve the margin. Although the study did not find IBTR occurring at a significantly higher rate compared to the non-ADH population, there were 3 IBTR observed in the ADH population. It would be important to know if these specimens contained ADH at the margin compared to the cases where IBTR did not occur.  Table 2 summarizes the findings from the aforementioned studies.

## 4. Conclusion

In conclusion, there is not enough evidence to direct reexcision when ADH is diagnosed at the margin of a BCS specimen for early stage breast cancer. The work of our paper did reveal that ADH at the margin of a BCS specimen is a controversial pathological diagnosis subject to large interobserver variability, and when this diagnosis is made in the setting of a known cancer, it may actually represent the peripheral extension of a neoplastic lesion. However, if ADH represents low grade DCIS, recent data supports that especially in ER positive tumors, when adjusting for hormonal and radiation adjuvant treatment, the significance associated with margin status and increased IBTR is less clinically likely. Further studies with longer followup and closer attention to ADH at the margin will be needed to answer this question directly. In the meantime, it is reasonable to consider reexcision of ADH at the surgical margin especially in the face of low grade DCIS, but further data is needed to provide more definitive recommendations. 

## Figures and Tables

**Table 1:** Interobserver variability among pathologists in cases of borderline ductal proliferative lesions.

Investigator	no. of pathologists/no. of slides reviewed	Concordance rates (%)
	6/24	5/5 agreed 0%
Rosai [21]		4/5 agreed 20%
		3/5 agreed 50%

	5/10	6/6 agreed 58%
Schnitt et al. [22]*		5/6 agreed 71%
		4/6 agreed 92%

	9/81	9/9 agreed 32%
Jain et al. [23]		8/9 agreed 52%
		7/9 agreed 63%

*Standardized criteria and formal education differentiating proliferative lesions provided to pathologist prior to reviewing slides.

**Table 2:** Atypical ductal hyperplasia (ADH) and ipsilateral breast tumor recurrence (IBTR).

Investigator	no. BCS specimens	no. lumpectomy + for ADH	IBTR ADH (+) no. (%)	IBTR in ADH (−) no. (%)	Median Followup (years)
Goldstein et al. [33]	94	54	**3/54 (5.6)**′	0/36 (0)′′	6.5
Fowble et al. [34]	460	99^†^	1/99 (1.0)	17/329 (5.2)	4.8
Greene et al. [35]	155*	87	0/87 (0%)**	1/68 (1.5)**	2.2

′ADH (+) at margin more recurrence than ADH (−) at margin *P* < 0.01.

′′Represents lesions negative for both ADH and COL.

^†^ADH involved somewhere in specimen, not specifically involving margin.

*Lumpectomy performed for ADH, no malignancy in original specimen.

**denotes malignancy development and not recurrence as initial lumpectomy was negative for malignancy.
